# An azido-oxazolidinone antibiotic for live bacterial cell imaging and generation of antibiotic variants

**DOI:** 10.1016/j.bmc.2014.05.054

**Published:** 2014-08-15

**Authors:** Wanida Phetsang, Mark A.T. Blaskovich, Mark S. Butler, Johnny X. Huang, Johannes Zuegg, Sreeman K. Mamidyala, Soumya Ramu, Angela M. Kavanagh, Matthew A. Cooper

**Affiliations:** Division of Chemistry and Structural Biology, Institute for Molecular Bioscience, The University of Queensland, Brisbane, Queensland 4072, Australia

**Keywords:** Antibiotics, Linezolid, Fluorescent probes, Bacteria, Click chemistry

## Abstract

An azide-functionalised analogue of the oxazolidinone antibiotic linezolid was synthesised and shown to retain antimicrobial activity. Using facile ‘click’ chemistry, this versatile intermediate can be further functionalised to explore antimicrobial structure–activity relationships or conjugated to fluorophores to generate fluorescent probes. Such probes can report bacteria and their location in a sample in real time. Modelling of the structures bound to the cognate 50S ribosome target demonstrates binding to the same site as linezolid is possible. The fluorescent probes were successfully used to image Gram-positive bacteria using confocal microscopy.

## Graphical abstract

**Figure:**
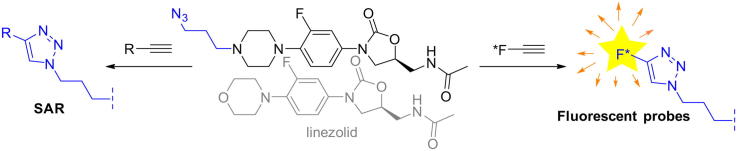

## 1 Introduction

Bacterial infection is a leading cause of death worldwide, and antimicrobial resistance has become one of the most serious public health concerns.^1^, ^2^ It has proven very difficult to identify new antibiotics, especially those with activity against drug-resistant bacteria, with very few new classes identified in the past 40 years.^3^, ^4^ The growing incidences of these drug-resistant ‘superbugs’ is fuelling a resurgence in microbiology research focused on the identification of new antibiotics, bacterial resistance mechanisms, new biological targets, and mode of action (MoA) studies.

**Figure 1:**
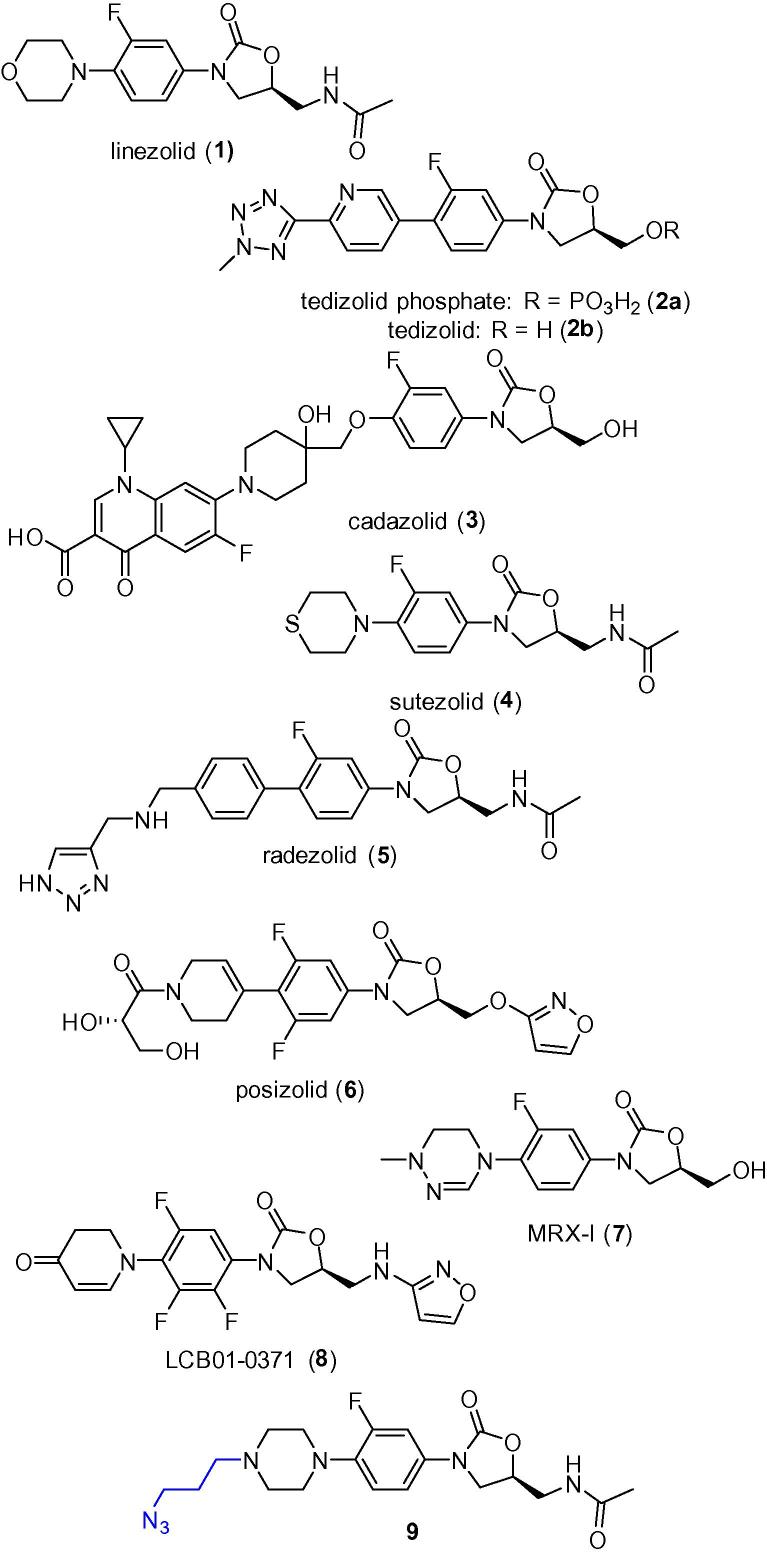
Oxazolidinone antibiotics in the clinic and the azide-functionalised linezolid analogue **9**.

Linezolid **1** (Zyvox®, Zyvoxid®, Zyvoxam®; Pfizer) was the first clinically used example of a novel class of chemically synthesized antimicrobial agents known as oxazolidinones (Fig. 1).^5^, ^6^ Developed in the 1990s and FDA approved in 2000, linezolid **1** is administered orally or intravenously for the treatment of Gram-positive infections. It is effective against methicillin-resistant *Staphylococcus aureus* (MRSA) and vancomycin resistant *Enterococcus* spp. (VRE). Resistance was noted within a year after its clinical introduction^7^, ^8^ and is still a concern.^9^ Linezolid **1** acts by inhibiting bacterial protein synthesis by targeting the ribosome, one of the most common antibiotic targets.^10^ Bacterial ribosomes are known as 70S ribosomes (composed of 30S and 50S subunits), whereas mammalian ribosomes are 80S structures (composed of 40S and 60S subunits).^10^ Antibiotics bind to bacterial ribosomes selectively because they differ from mammalian ribosomes. Oxazolidinone antibiotics bind to the 50S subunit and block formation of the 70S initiation complex by preventing assembly of the *N*-formyl-methionyl-tRNA–ribosome–mRNA ternary complex.^11^, ^12^ In addition, they interfere with translocation of peptidyl-tRNA from the A site to the P site.^13^ Resistance to linezolid **1** is associated with modifications of its binding site on the ribosome.^14^ Resistance to oxazolidinones also involves mutation of 23S rRNA, resulting in decreased binding affinity.^15^

A number of new oxazolidinone derivatives are in clinical development (Fig. 1).^4^ Tedizolid phosphate **2a** (Sivextro, torezolid phosphate, TR-701, DA-7218; Cubist/Trius Therapeutics) is a prodrug that has completed phase-III trials and a New Drug Application (NDA) has been filed in the US and Marketing Authorization Application (MAA) for Europe, with FDA approval granted in June 2014.^16^, ^17^, ^18^ Dephosphorylation in vivo unmasks tedizolid **2b**, which is active against linezolid resistant strains.^19^ Cadazolid **3** (ACT-179811; Actelion Pharmaceuticals) is a quinolonyl-oxazolidinone chimeric antibiotic that recently started a phase-III trial for the treatment of patients with *Clostridium difficile* infection (CDI).^20^, ^21^ Four oxazolidinones have completed or are in phase-II trials. Sutezolid **4** (PNU-100480, PF-02341272; Sequella/Pfizer) is a close analogue of linezolid **1** that was optimized for activity against tuberculosis.^22^, ^23^, ^24^, ^25^, ^26^ Radezolid **5** (RX-1741; Melinta Therapeutics, formally RibX) is similar in structure to tedizolid, and has activity against both Gram-positive and some Gram-negatives bacteria.^27^, ^28^, ^29^, ^30^, ^31^, ^32^ Posizolid **6** (AZD5847, AZD2563; AstraZeneca) is another oxazolidinone being assessed for treatment of tuberculosis, although it was originally developed as a broad spectrum Gram-positive antibiotic.^33^, ^34^, ^35^ MRX-I (MicuRx) **7** recently started Phase II trials in China.^36^, ^37^ LCB01-0371 **8** (LegoChem Biosciences)^38^ is currently being evaluated in phase-I trials.

The development of new antibiotics and alternative strategies to combat antibiotic-resistant bacteria is aided by an improved understanding of antibiotic interactions with bacteria and bacterial cellular complexity. Fluorescent imaging has been used to improve our comprehension. For example, the peptidoglycan layer (PG) plays an important role in cell wall structure. Vancomycin fluorescent probes binding to peptidoglycan precursors were used to stain *Bacillus subtilis*, and indicated that although bacterial actin homologues played an important role in cell shape determination, other proteins controlled the spatial localization of the biosynthetic complexes responsible for new PG synthesis.^39^ In another study a vancomycin probe was used as an optical imaging tool for detecting infecting bacteria in vivo.^40^ Vancomycin linked with a near-infrared (NIR) probe binds to Gram-positive bacteria, allowing invasive infection to be detected by NIR optical imaging. Boron-dipyrromethene (BODIPY)-labeled daptomycin (BDP-DAP) was used to determine a novel resistance mechanism of vancomycin-resistant enterococci (VRE) to cationic antimicrobial peptides.^41^ From this study, VRE was shown to resist DAP-elicited cell membrane damage by diverting the antibiotic away from its principal target to other distinct cell membrane regions.

The Cu-catalysed azide–alkyne cycloaddition (an azide–alkyne version of the Huisgen 1,3-dipolar cycloaddition) is a useful reaction for derivatising azide/alkyne substituents under mild conditions.^42^, ^43^, ^44^ The ‘click’ generated triazole is very useful in biological studies and medicinal chemistry due to the favourable physicochemical properties of the triazole ring.^45^ Triazoles are stable to reductive and oxidative reactions and acidic and basic hydrolysis reactions, under conditions where amides can be hydrolytically cleaved.^46^ Furthermore, the aromatic structure of triazoles resists enzymatic degradation and also participates in hydrogen bond formations and π-stacking interactions.^47^, ^48^

In this study, we prepare an azide-functionalised oxazolidinone antibiotic, and use it as a convenient intermediate in azide–alkyne click reactions to rapidly generate modified analogues, including fluorescent probes.

## 2 Materials and methods

### 2.1 Synthesis of azide-derivatised linezolid

**Scheme 1:**
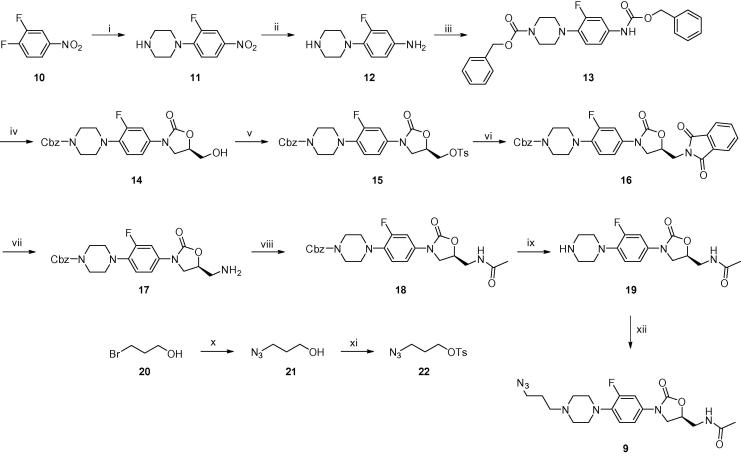
Synthesis of azide derivatised oxazolidinone antibiotic **9**, following literature procedure^49^ for **19.** Reagents and conditions: (i) piperazine, CH_3_CN, reflux; (ii) H_2_, 10% Pd/C, THF; (iii) benzyloxycarbonyl chloride (Cbz-Cl), Na_2_CO_3_, acetone/H_2_O; (iv) *n*-BuLi, THF, −78 °C, (*R*)-glycidyl butyrate; (v) TsCl, NEt_3_, CH_2_Cl_2_; (vi) potassium phthalimide, CH_3_CN, H_2_O, reflux; (vii) aqueous MeNH_2_, EtOH, reflux; (viii) Ac_2_O, pyridine; (ix) H_2_, 10% Pd/C, MeOH/CH_2_Cl_2_; (x) NaN_3_, THF/H_2_O, reflux; (xi) TsCl, NEt_3_, CH_2_Cl_2_, 0 °C–rt; (xii) **22**, NEt_3_, NaI, EtOH, reflux.

The intermediate linezolid derivative **19** (Scheme 1) was synthesised as described in the literature,^49^ except that a tosylate was used instead of a mesylate for the conversion of **15** to **16**. In short, 3,4-difluoronitrobenzene **10** was treated with piperazine, selectively resulting in *p*-substituted nitrobenzene **11**. Catalytic reduction of **11** and subsequent acylation of amine **12** with benzylchloroformate gave protected carbamate **13**. Reaction of **13** with (*R*)-glycidyl butyrate in THF at −78 °C in the presence of *n*-BuLi provided 5-(*R*)-hydroxymethyl-2-oxazolidinone **14**. To form the desired oxazolidinone ring it was essential to use a lithium counter ion in the base for regiochemical control. Sodium and potassium bases generate 5-hydroxymethyl-2-oxazolidinone as a major by-product.^50^ Treatment of compound **14** with *p*-toluenesulfonyl chloride at 0 °C in the present of triethylamine gave the tosylated product **15**, which was displaced by phthalimide to produce compound **16**. Deprotection of phthalimide with methylamine generated the free amine **17** which was acetylated with acetic anhydride to provide **18**. Catalytic deprotection of the Cbz group with palladium on activated carbon gave the intermediate linezolid derivative **19**.

A linker **22** containing the azido group was synthesised in 2 steps from 3-bromo-1-propanol **20**, which was treated with sodium azide in THF/H_2_O. The reaction mixture was refluxed with stirring for 16 h to yield 3-azido-1-propanol **21**.^51^ Treatment of with *p*-toluenesulfonyl chloride at 0 °C in the present of triethylamine gave the tosylated product **22**.

Finally, the secondary amine contained in **19** was alkylated with azide linker **22** to yield azide-derivatised linezolid analogue **9** in good yield.

### 2.2 Synthesis of alkyne-derivatised fluorophores

**Scheme 2:**
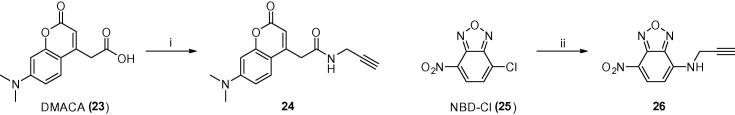
Synthesis of alkyne-functionalised fluorophores. Reagents and conditions: (i) propargylamine, HATU, DIPEA, DMF rt; (ii) propargylamine, Cs_2_CO_3_, THF.

Two fluorophores, 7-(dimethylamino)-coumarin-4-acetic acid (DMACA, prepared according to literature procedures^52^, ^53^) and 7-nitrobenzofurazan (NBD, Sigma–Aldrich), were functionalized with an alkyne substituent (Scheme 2) so that they could be coupled to the linezolid–azide analogue **9** by click chemistry. DMACA **23** was reacted with propargylamine in the presence of HATU as coupling agent to give DMACA linked alkyne **24**. NBD linked alkyne **26** was prepared by a substitution reaction from NBD-Cl (4-chloro-7-nitrobenzofurazan) **25** by an improved method based on that previously reported in the literature.^54^ The use of Cs_2_CO_3_ in THF for the substitution gave improved yields compared to aqueous NaHCO_3_ in MeOH.

### 2.3 Click chemistry of azide-derivatised oxazolidinone

**Scheme 3:**
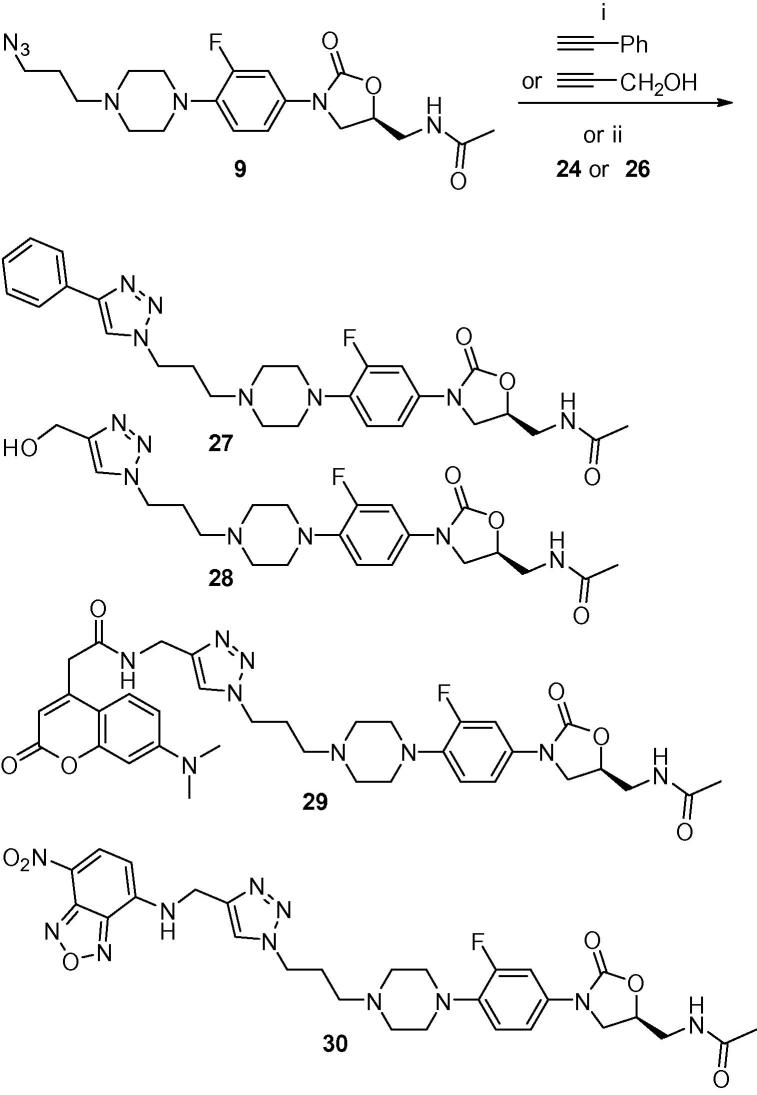
‘Click’ reaction to generate functionalized oxazolidinone antibiotics and fluorescent probes. Reagents and conditions: (i) sodium ascorbate, CuSO_4_, MeOH, rt; (ii) sodium ascorbate, CuSO_4_, MeOH, DMF, 50 °C.

To demonstrate the potential of the azide-functionalised oxazolidinone to generate analogues to explore antimicrobial SAR and produce fluorescent probes, two different alkynes and the two alkyne derivatised fluorophores were reacted with the azide. Linezolid azide analogue **9** was reacted with propargyl alcohol, phenylacetylene, and alkyne fluorophores **24** and **26** in the presence of copper sulfate and sodium ascorbate to provide the corresponding triazole derivatives **27**, **28**, **29**, and **30**, respectively (Scheme 3). The cycloaddition reactions of the alkyne-fluorophores and azide **9** at room temperature were found to be very slow, hence were conducted at 50 °C with completion after 16 h. All products were purified by reverse-phase HPLC.

### 2.4 MIC assays

Compounds were tested for antimicrobial activity against twelve Gram-positive bacterial strains: *Enterococcus faecalis* (VanA clinical isolate), *Enterococcus faecium* (MDR Van A ATCC 51559), *Streptococcus pneumoniae* (MDR ATCC 700677), *Staphylococcus aureus* (MRSA ATCC 43300, MRSA clinical isolate, MRSA daptomycin resistant clinical isolate, GISA NRS 1, GISA NRS 17, VRSA NARSA VRS1, VRSA NARSA VRS4 and VRSA NARSA VRS10). All experiments were performed in duplicate with vancomycin, and linezolid used as positive controls (see Table 1). Positive growth control rows of bacteria and DMSO + bacteria as well as a negative control row of only media were included for every plate.

**Table 1:** MIC (minimum inhibitory concentrations) of oxazolidinone derivatives against Gram-positive bacteria

Compound	MIC (μg/mL)										
	*Staphylococcus aureus*								*Streptococcus pneumoniae*	*Enterococcus faecalis*	*Enterococcus faecium*
	MRSA ATCC 43300	MRSA Clin. Isol.	DaptRs Clin. Isol.	GISA NRS1	GISA NRS 17.	VRSA NARSA VRS1	VRSA NARSA VRS4	VRSA NARSA VRS10	MDR ATCC 700677	Van A Clin. Isol	Van A ATCC 51559
Vancomycin	1	1	2	8	4	>64	32	1	1	>64	>64
Linezolid **1**	1	1	1	1	1	1	2	2	1	2	2
19	8	2	2	4	4	4	32	8	4	8	0.5
**9** Lz-N3	2	2	2	2	2	2	2	2	4	4	4
**27** Lz-tz-Ph	64	32	>64	16	16	16	64	32	64	16	16
**28** Lz-tz-CH2OH	4	2	4	2	4	4	8	8	4	32	2
**29** Lz-tz-DMACA	>64	64	>64	>64	>64	64	>64	64	64	64	32
**30** Lz-tz-NBD	16	8	8	8	16	16	32	32	4	32	16

Clin. Isol. = clinical isolate; DaptRs = daptomycin resistant; MRSA = methicillin resistant *S. aureus*, GISA = glycopeptide insensitive *S. aureus*, MDR = multidrug resistant, VRSA = vancomycin resistant *S. aureus*.

### 2.5 Fluorescent properties of probes

**Figure 2:**
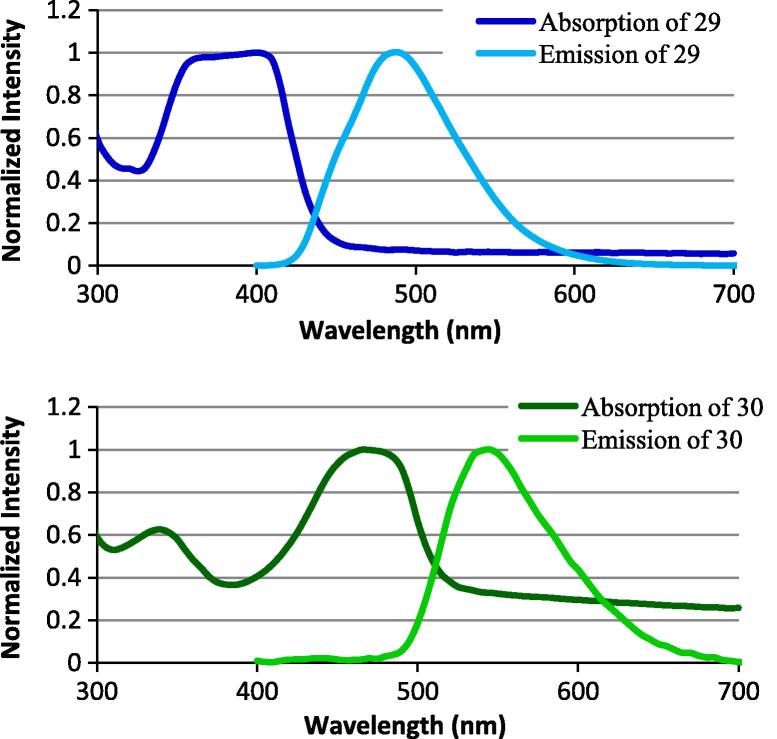
Excitation/emission of fluorophore probes **29** and **30**.

The fluorescent spectra of the two oxazolidinone-fluorescent probe conjugates **29** and **30** were measured (Fig. 2). Note that the DMACA emission peak of **29** (*λ*_max_ = 490 nm) overlaps with the NBD absorption peak of **30** (*λ*_max_ = 475 nM).

### 2.6 Fluorescent imaging of bacteria

The ability of the fluorescent probes to label bacteria was examined using log phase cultures of two *S. aureus* strains (an MRSA clinical isolate and a VISA NARSA VRS3b) and an *Enterococcus faecium* strain (MDR Van A ATCC 51559). Probes **29** and **30** were incubated with the bacteria for one hour at a concentration of 64 μg/mL, after which the bacteria were smeared onto glass slides. After fixation, washing and mounting steps, fluorescent images of the slides were obtained using a confocal microscope (Zeiss LSM 510 META).

### 2.7 Docking studies of oxazolidinone derivatives

The derivatives were modelled into the crystal structure of native *Deinococcus radiodurans* large ribosomal subunit (D50S) bound with linezolid (Pdb: 3DLL^55^) using Schrödinger software package and its Induced Fit Docking module (Induced Fit Docking protocol 2013-2, Glide version 5.9, Prime version 3.2, Schrödinger, LLC, New York, NY, 2013), to account for the reported structural flexibility of the peptidyl transferase centre.

## 3 Results and discussion

### 3.1 Design of azide-derivatised linezolid analogue

**Figure 3:**
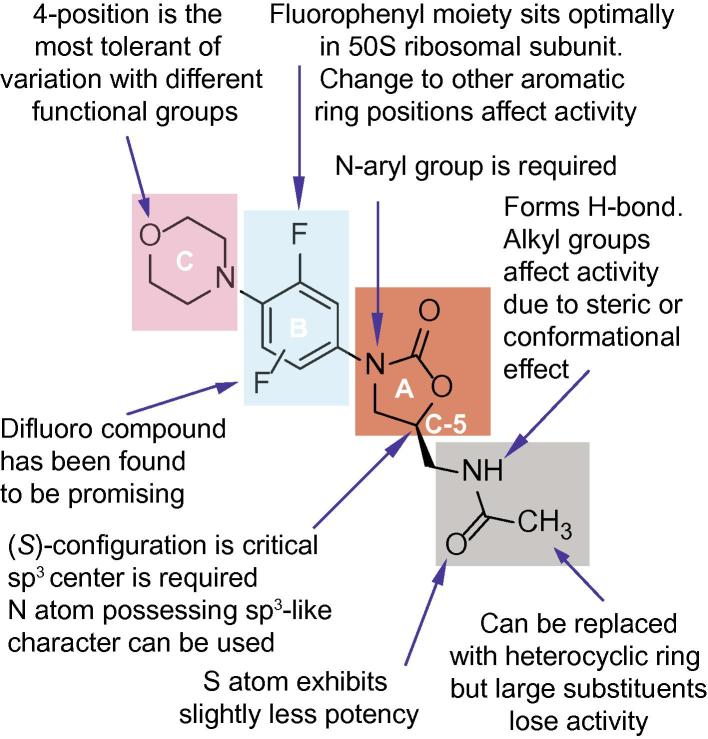
Known structure–activity relationships of oxazolidinone antibiotics.

The oxazolidinone antibiotic linezolid **1** possesses 4 key structural elements; the A-, B-, and C-rings, and the C-5 position of the A-ring (Fig. 3). Modification of linezolid’s A-ring, B-ring, and C-5 position influence its antimicrobial activity. Most modified oxazolidinone antibiotics keep the B-ring to maintain potent activity. However, the 4′-position of the C-ring was tolerant to alteration and does not show significant loss of activity after replacement with different functional groups.^12^, ^56^, ^57^, ^58^ This correlates with structural analysis of different ribosomal subunits in comparison with the binding mode of linezolid^55^, ^59^ in which the nucleotide U2585 at the peptidyltransferase centre shows considerable conformational flexibility, and is able to accommodate large substituents at the 4′-position of linezolid. Therefore, an azide-derivatized linezolid **9** was designed to explore the 4′-position of the C-ring.

### 3.2 ‘Click’ chemistry and antimicrobial activity of conjugates

Four different alkynes were successfully employed for proof-of-principle Cu-catalysed azide–alkyne cycloadditions with the azide-derivatised oxazolidinone, generating the triazole-linked conjugates. The reactions proceeded in good yield to give the desired products. As expected, the reaction was tolerant of a range of functionalities on both the azide and alkyne components.

**Figure 4:**
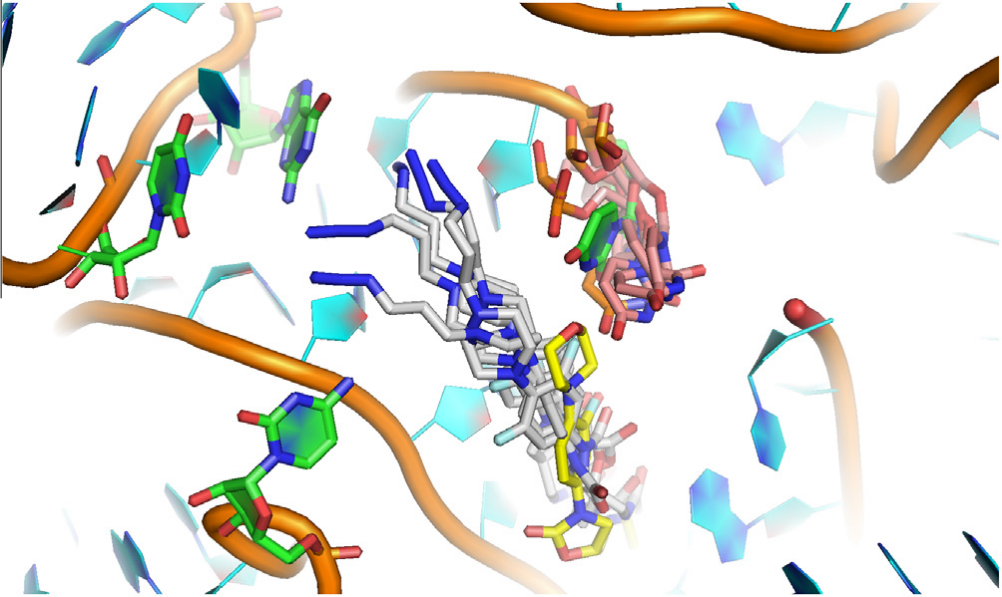
In silico docking studies of the azide-functionalised oxazolidinone **9** within the binding site of linezolid in ribosomal subunit 50S of *D. radiodurans*. Crystal structure 3DLL shown as cartoon, with key nucleotides in green and linezolid in yellow. Docked structure of **9** shown in grey with conformational variations of nucleotide U2585 in orange.

The linezolid/sutezolid analogue **19**, replacing the morpholine oxygen with a nitrogen, consistently lost approximately 4-fold potency, with the exception of multidrug resistant *E. faecium* ATCC 51559, where potency was gained. Substitution with the azidopropyl substituent, in the azide-functionalised linezolid intermediate **9**, gave a compound that retained similar activity to linezolid against all strains tested (MIC 2 to 4 μg/mL). Triazole formation with phenylacetylene or propargyl alcohol resulted in significantly different activity, with the phenyl-substituted triazole **27** much less active (MIC 32 to >64 μg/mL) than the hydroxymethyl derivative **28** (MIC 2 to 32 μg/mL). The linezolid-DMACA probe **29** lost most antimicrobial activity (MIC 32 to >64 μg/mL) whereas the linezolid-NBD probe **30** retained some activity (4 to 32 μg/mL) against most strains tested. From the in silico docking studies the azide-functionalised oxazolidinone **9** was able to fit into a similar position observed for linezolid in the crystal structure of ribosomal subunit 50S of *D. radiodurans* (Fig. 4).^55^ Due to the extended size of the molecule an induced fit workflow had to be applied in order to accommodate the molecule. As reported in other crystal structures, the nucleotide U2585 was able to adopt a variation of conformations, while the piperazine azide moiety oriented itself along the P-site. The fact that some activity was retained for the fluorescent probes was promising, as it indicated that some compound was penetrating the bacterial membrane, and for staining of live bacteria sub-MIC concentrations could be used.

### 3.3 Fluorophore-derivatised oxazolidinone antibiotics

**Figure 5:**
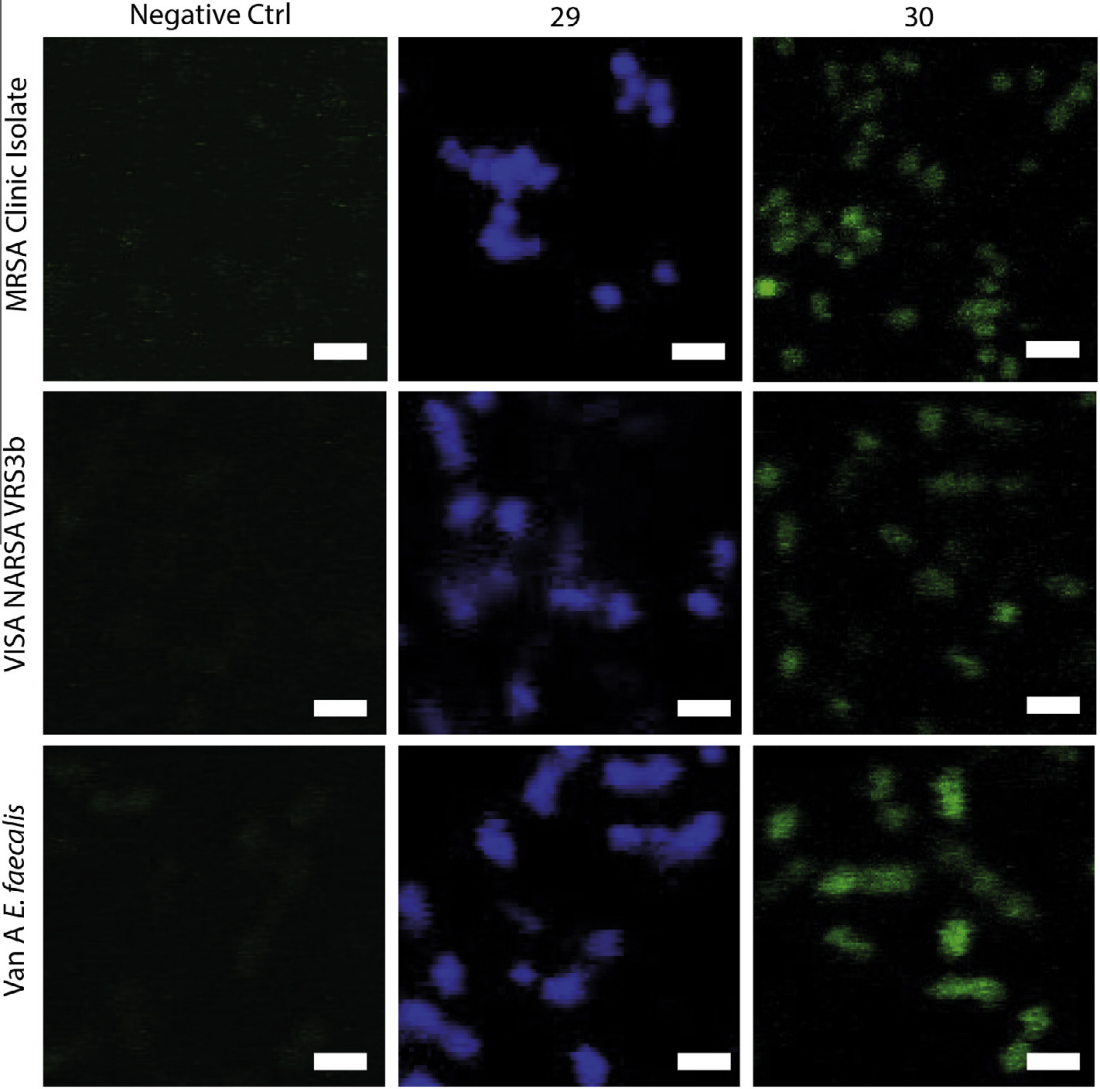
Labelling of Gram positive bacteria with fluorescent probes **29** and **30**. Negative controls: bacteria only. Bars, 2 μm.

Two different fluorophores, 7-(dimethylamino)-coumarin-4-acetic acid (DMACA) and 7-nitrobenzofurazan (NBD) were selected as initial fluorophores due to their low molecular size compared to most other fluorophores (such as rhodamines and fluoresceins), increasing both the potential for cellular penetration of the antibiotic-fluorophore probes, and the likelihood that they could be accommodated within the linezolid binding site. They also have differing fluorescence colours (blue and green, respectively), with an emission/excitation overlap that may be useful for FRET studies. The alkyne-derivatised fluorophores were readily coupled with the azide-oxazolidinone **9** to generate fluorescent probes **29** and **30**. Incubation of these probes with Gram-positive bacteria resulted in selective staining of the bacteria cells (Fig. 5), with the staining pattern consistent with internalization when compared to other fluorophore probes known to stain the cell surface (e.g., similar to internal staining pattern of DAPI, 4′,6-diamido-2-phenylindole, which binds internal nucleic acids, but not like the membrane binding dye FM4-64, *N*-(3-triethylammoniumpropyl)-4-(6-(4-(diethylamino)phenyl)-hexa-trienyl)pyridinium dibromide^60^). The probes labelled both *S. aureus* and *E. faecium* despite relatively poor MIC values.

## 4 Conclusion

In conclusion, we have demonstrated that a morpholine-to-piperidine analogue of the oxazolidinone antibiotic linezolid can be alkylated with azidopropane to produce an azide-modified derivative that retains the antibacterial activity of linezolid. This versatile intermediate can be applied to rapidly assess structure–activity relationships using Cu-catalysed azide–alkyne cycloadditions with substituted alkynes, and to easily conjugate fluorophores with different physicochemical and photochemical properties. The oxazolidinone-fluorophore probes successfully labelled a number of different types of Gram-positive bacteria. This approach allows for a rapid ‘mix and match’, with alternate fluorophore colours readily attached to the derivatised antibiotic in a single step. Future studies will examine the utility of these probes in bacterial detection systems, and their ability to detect internal bacterial structures when combined with high resolution imaging, such as 3D structured illumination microscopy (3D-SIM-Microscopy).^61^ The azide-modified oxazolidinone will also be combined with other functionalized antibiotics to rapidly generate hybrid antibiotics similar to cadazolid **3**.

## 5 Experimental

### 5.1 Chemistry

#### 5.1.1 General

All materials, unless otherwise noted, were obtained from commercial suppliers and used without further purification. Non-aqueous reactions were conducted under an inert atmosphere of nitrogen. Reactions were monitored by thin layer chromatography (TLC). Analytical TLC was performed on Merck TLC alumina sheets pre-coated with Silica Gel 60 F254, and compounds were visualized using UV lamp and appropriate TLC stains. Column chromatography was performed using silica gel 60 (0.063–0.200 mm), 70–230 mesh ASTM. Biotage Isolera and Grace Reveleris chromatography systems were used for compound purification. ^1^H (600 MHz) and ^13^C (125 MHz) NMR spectra were obtained using a Bruker Avance-600 spectrometer equipped with a TXI cryoprobe. Chemical shifts are reported relative to the residual solvent signals in parts per million (*δ*) (CDCl_3_: ^1^H: *δ* 7.27, ^13^C: *δ* 77.2; CD_3_OD: ^1^H: *δ* 3.30, ^13^C: *δ* 49.5; DMSO-*d*_6_: ^1^H: *δ* 2.50, ^13^C: *δ* 39.5). High resolution mass spectrometry (HRMS) was performed on a Bruker Micro TOF mass spectrometer using (+)-ESI calibrated to NH_4_OAc.

#### 5.1.2 Synthesis of linezolid analogue **19**

Linezolid analogue **19** was synthesized by a slight modification (mesylate displacement replaced with tosylate) of a literature method^49^ as shown in Scheme 1.

#### 5.1.3 3-Azidopropyl 4-methylbenzenesulfonate **22**

The mixture of 3-bromo-1-propanol **20** (1.0 g, 7.19 mmol) and sodium azide (1.4 g, 21.58 mmol) in THF/H_2_O (20:5 mL) was stirred at 80 °C for 16 h. The reaction mixture was extracted with CH_2_Cl_2_, dried over MgSO_4_, and concentrated under reduced pressure to give compound **21** (0.59 g, 81%) as an oil which was used for the next reaction without further purification.

To the solution of 3-azidopropan-1-ol **21** (1.47 g, 14.55 mmol) in CH_2_Cl_2_ was added *p*-toluenesulfonyl chloride (3.05 g, 16.01 mmol). The reaction mixture was stirred at rt overnight. The reaction was checked by TLC for completion. The resulting residue was diluted with water and extracted with CH_2_Cl_2_, dried over MgSO_4_, and concentrated under reduced pressure. Purification by column chromatography (silica gel, 50% EtOAc/Hexane) gave compound **22** (2.58 g, 69%) as an oil. ^1^H NMR (600 MHz, CDCl_3_): *δ* 7.76 (d, *J *= 8.2 Hz, 2H), 7.33 (d, *J *= 8.4 Hz, 2H), 4.07 (t, *J *= 5.8 Hz, 2H), 3.34 (t, *J *= 6.5 Hz, 2H), 2.42 (s, 3H), 1.85 (quin, *J *= 6.2 Hz, 2H); ^13^C NMR (125 MHz, CDCl_3_): *δ* 145.2, 132.8, 130.1, 128.0, 67.2, 47.4, 28.6, 21.8; (+)-ESI-HRMS calc for C_10_H_13_N_3_NaO_3_S [M+Na^+^]^+^: 278.0575, found 278.0580.

#### 5.1.4 (*S*)-*N*-((3-(4-(4-(3-Azidopropyl)piperazin-1-yl)-3-fluorophenyl)-2-oxooxazolidin-5-yl)methyl)-acetamide **9**

To the mixture of **19** (100 mg, 0.30 mmol), **22**, and NaI (10 mol %, 5 mg, 0.003 mmol) in EtOH (40 mL), NEt_3_ was added. The mixture was stirred under reflux for 6 h and then concentrated under reduced pressure. Purification by column chromatography (Grace MPLC; C18 reverse phase with eluents 0.1% TFA in MeOH/ 0.1% TFA in H_2_O) gave compound **9** (46 mg, 37%) as white solid. ^1^H NMR (600 MHz, CD_3_OD): *δ* 7.48 (dd, *J *= 14.7, 2.5 Hz, 1H), 7.16 (dd, *J *= 8.8, 2.6 Hz, 1H), 7.05 (t, *J *= 9.4 Hz, 1H), 4.80 (m, 1H), 4.11 (t, *J *= 9.0 Hz, 1H), 3.80–3.77 (m, 1H), 3.55 (d, *J *= 5.0 Hz, 2H), 3.40 (t, *J *= 6.6 Hz, 2H), 3.10 (br s, 4H), 2.69 (s, 4H), 2.55 (t, *J *= 7.5 Hz, 2H), 1.96 (s, 3H), 1.85–1.80 (m, 2H); ^13^C NMR (125 MHz, CD_3_OD): *δ* 174.2, 156.9 (d, *J *= 245.1 Hz), 156.8, 137.6 (d, *J *= 9.1 Hz), 135.1 (d, *J *= 10.5 Hz), 120.5 (d, *J *= 3.9 Hz), 115.6 (d, *J *= 2.9 Hz), 108.6 (d, *J *= 26.5 Hz), 73.6, 56.7, 54.4, 51.6 (d, *J *= 2.6 Hz, 2C), 50.7, 49.3, 43.3, 27.1, 22.6; (+)-ESI-HRMS calc for C_19_H_27_FN_7_O_3_ [M+H]^+^: 420.2159, found 420.2174.

#### 5.1.5 2-(7-(Dimethylamino)-2-oxo-2*H*-chromen-4-yl)-*N*-(prop-2-yn-1-yl)acetamide **24**

To a solution of the 2-(7-(dimethylamino)-2-oxo-2*H*-chromen-4-yl)acetic acid **23** (0.3 g, 1.21 mmol) in DMF (5 mL) was added HATU in DMF (5 mL) followed by DIPEA (386 μL), and propargylamine (71 μL, 1.1 mmol). The solution was stirred at rt overnight. The reaction was evaporated under reduced pressure to remove DMF. The residue was diluted with water and extracted with ethyl acetate, dried over MgSO_4_, and concentrated under reduced pressure. The crude compound was recrystallized in CH_2_Cl_2_. The solid was filtrated and washed with CH_2_Cl_2_ to give pure compound **24** (0.149 g, 48%) as a green solid. ^1^H NMR (600 MHz, DMSO-*d*_6_): *δ* 8.65 (t, *J *= 5.4 Hz, 1H), 7.52 (d, *J *= 9.0 Hz, 1H), 6.72 (dd, *J *= 9.1, 2.6 Hz, 1H), 6.55 (d, *J *= 2.6 Hz, 1H), 6.00 (s, 1H), 3.88–3.87 (m, 2H), 3.62 (s, 2H), 3.13 (t, *J *= 2.5 Hz, 1H), 3.01 (s, 6H); ^13^C NMR (125 MHz, DMSO-*d*_6_): *δ* 167.7, 160.7, 155.4, 152.9, 151.0, 126.0, 109.4, 109.1, 108.1, 97.5, 80.9, 73.3, 39.7, 38.4, 28.2; (+)-ESI-HRMS calc for C_32_H_32_N_4_NaO_6_ [2M+Na]^+^: 591.2220, found 591.2190.

#### 5.1.6 7-Nitro-*N*-(prop-2-yn-1-yl)benzo[*c*][1,2,5]-oxadiazol-4-amine **26**

To a solution of 4-chloro-7-nitrobenzo[*c*][1,2,5]oxadiazole (300 mg, 1.5 mmol) in THF (10 mL) was added a solution of propargyl amine (110 μL, 1.65 mmol), Cs_2_CO_3_ (480 mg, 1.5 mmol). The reaction mixture was stirred at 50 °C for 4 h. After completion of the reaction, the reaction mixture was diluted with EtOAc (50 mL), washed with H_2_O (30 mL), brine (30 mL). The organic phase was separated, dried (MgSO_4_), and evaporated to give the residue. The residue was purified by Si column chromatography (petroleum ether/EtOAc, 7:3) to afford **26** (240 mg, 75%). ^1^H NMR (600 MHz, CDCl_3_): *δ* 8.54 (d, *J* = 8.4 Hz, 1H), 6.35 (d, *J* = 8.4 Hz, 1H), 6.32 (s, 1H, NH), 4.3 (dd, *J* = 2.4, 5.6 Hz, 2H), 2.44 (t, *J* = 2.4 Hz, 1H).

#### 5.1.7 (*S*)-*N*-((3-(3-Fluoro-4-(4-(3-(4-phenyl-1*H*-1,2,3-triazol-1-yl)propyl)piperazin-1-yl)phenyl)-2-oxooxazolidin-5-yl)methyl)acetamide **27**

To a solution of **9** (18.9 mg, 0.045 mmol) and phenylacetylene (5 μL, 0.045 mmol) in MeOH (4 mL) was added a solution of CuSO_4_ (5 mol %, 0.56 mg, 0.0023 mmol) and treated with aqueous sodium ascorbate (10 mol %, 0.89 mg, 0.0045 mmol). The reaction was stirred vigorously for 16 h. The reaction was concentrated under reduced pressure. Purification by column chromatography (Grace MPLC; C18 reverse phase with eluents 0.1% TFA in MeOH/0.1% TFA in H_2_O) gave compound **27** (5.66 mg, 24%) as a white solid. ^1^H NMR (600 MHz, DMSO-*d*_6_): *δ* 8.62 (s, 1H), 8.24 (t, *J *= 5.8 Hz, 1H), 7.86–7.84 (m, 2H), 7.51 (dd, *J *= 14.7, 2.5 Hz, 1H), 7.48–7.45 (m, 2H), 7.35 (tt, *J *= 7.4, 1.2 Hz, 1H), 7.21 (dd, *J *= 8.8, 2.2 Hz, 1H), 7.14 (t, *J *= 9.4 Hz, 1H), 4.73–4.69 (m, 1H), 4.54 (t, *J *= 6.7 Hz, 2H), 4.08 (t, *J *= 9.0 Hz, 1H), 3.71–3.69 (m, 1H), 3.61 (br s, 2H), 3.45–3.39 (m, 4H), 3.24 (br s, 4H), 3.01 (br s, 2H), 2.34 (br s, 2H), 1.83 (s, 3H); ^13^C NMR (125 MHz, DMSO-*d*_6_): *δ* 170.0, 154.5 (d, *J *= 243.6 Hz), 154.0, 146.4, 134.3 (d, *J *= 10.2 Hz), 130.7, 128.9, 127.9, 125.1, 121.6, 119.9 (d, *J *= 3.4 Hz), 114.1 (d, *J *= 3.1 Hz), 106.6 (d, *J *= 26.0 Hz), 71.6, 53.0, 51.2, 47.4, 47.3, 46.9, 41.2, 24.3, 22.4; (+)-ESI-HRMS calc for C_27_H_33_FN_7_O_3_ [M+H]^+^: 522.2629, found 522.2627.

#### 5.1.8 (*S*)-*N*-((3-(3-Fluoro-4-(4-(3-(4-(hydroxymethyl)-1*H*-1,2,3-triazol-1-yl)propyl)piper-azin-1-yl)phenyl)-2-oxooxazolidin-5-yl)methyl)acetamide **28**

To a solution of **9** (24 mg, 0.057 mmol) and propargyl alcohol (3.2 μL, 0.057 mmol) in MeOH (4 mL) was added a solution of CuSO_4_ (5 mol %, 0.71 mg, 0.0029 mmol) and treated with aqueous sodium ascorbate (10 mol %, 1.13 mg, 0.0057 mmol). The reaction was stirred vigorously for 16 h. The reaction was concentrated under reduced pressure. Purification by column chromatography (Grace MPLC; C18 reverse phase with eluents 0.1% TFA in MeOH/0.1% TFA in H_2_O) gave compound **28** (13.3 mg, 49%) as a white solid. ^1^H NMR (600 MHz, DMSO-*d*_6_): *δ* 8.24 (t, *J *= 5.8 Hz, 1H), 8.02 (s, 1H), 7.51 (dd, *J *= 14.7, 2.5 Hz, 1H), 7.20 (dd, *J *= 8.9, 2.2 Hz, 1H), 7.13 (t, *J *= 9.2 Hz, 1H), 4.73–4.69 (m, 1H), 4.53 (s, 2H), 4.45 (t, *J *= 6.8 Hz, 2H), 4.08 (t, *J *= 9.0 Hz, 1H), 3.71–3.69 (m, 1H), 3.59 (br s, 2H), 3.41–3.36 (m, 4H), 3.19 (br s, 4H), 3.01 (br s, 2H), 2.26, (br s, 2H), 1.83 (s, 3H); ^13^C NMR (125 MHz, CD_3_OD): *δ* 174.3, 157.1 (d, *J *= 245.4 Hz), 156.7, 136.4 (d, *J *= 10.6 Hz), 135.7 (d, *J *= 9.5 Hz), 121.3 (d, *J *= 3.2 Hz), 115.6 (d, *J *= 2.9 Hz), 108.6 (d, *J *= 26.3 Hz), 73.7, 56.5, 55.4, 53.6, 49.2, 48.4, 43.3, 25.9, 22.6; ^13^C NMR (125 MHz, DMSO-*d*_6_): *δ* 170.0, 154.5 (d, *J *= 244.3 Hz), 154.0, 148.1, 122.8, 119.92, 114.1 (d, *J *= 3.0 Hz), 106.6 (d, *J *= 26.0 Hz), 71.6, 55.1, 53.0, 51.2, 47.3, 46.6, 41.4, 22.4; (+)-ESI-HRMS calc for C_22_H_31_FN_7_O_4_ [M+H]^+^: 476.2422, found 476.2419.

#### 5.1.9 (*S*)-*N*-((1-(3-(4-(4-(5-(Acetamidomethyl)-2-oxooxazolidin-3-yl)-2-fluorophenyl)piperazin-1-yl)propyl)-1*H*-1,2,3-triazol-4-yl)methyl)-2-(7-(dimethylamino)-2-oxo-2*H*-chromen-4-yl)acetamide **29**

To a solution of azide **9** (50 mg, 0.12 mmol) and DMACA fluorophore **24** (34 mg 0.12 mmol) in DMF (4 mL) was added a solution of CuSO_4_ (5 mol %, 1.5 mg, 0.006 mmol) and treated with aqueous sodium ascorbate (10 mol %, 2.4 mg, 0.012 mmol). The reaction was stirred vigorously at 50 °C for 16 h. Purification by column chromatography (Grace MPLC; C18 reverse phase with eluents 0.1% TFA in ACN/0.1% TFA in H_2_O) gave compound **54** (61.5 mg, 79%) as a green solid. ^1^H NMR (600 MHz, DMSO-*d*_6_): *δ* 8.76 (t, *J *= 5.7 Hz, 1H), 8.24 (t, *J *= 5.9 Hz, 1H), 7.98 (s, 1H), 7.54–7.50 (m, 2H), 7.21 (dd, *J *= 8.8, 2.2 Hz, 1H), 7.14 (t, *J *= 9.4 Hz, 1H), 6.71 (dd, *J *= 9.1, 2.5 Hz, 1H), 6.55 (d, *J *= 2.5 Hz, 1H), 5.99 (s, 1H), 4.73–4.69 (m, 1H), 4.44 (t, *J *= 6.9 Hz, 2H), 4.33 (d, *J *= 5.7 Hz, 2H), 4.08 (t, *J *= 9.0 Hz, 1H), 3.71–2.20 (m, 11H), 3.19 (br s, 4H), 3.02 (s, 6H), 2.26 (quin, *J *= 6.9 Hz, 2H), 1.83 (s, 3H); ^1^H NMR (600 MHz, CD_3_OD): *δ* 7.79 (s, 1H), 7.53–7.51 (m, 2H), 7.17 (d, *J *= 8.8 Hz, 1H), 7.07 (t, *J *= 9.2 Hz, 1H), 6.72–6.71 (m, 1H), 6.50 (s, 1H), 5.95 (s, 1H), 4.79–4.75 (m, 1H), 4.53 (t, *J *= 6.6 Hz, 2H), 4.46 (s, 2H), 4.10 (t, *J *= 9.1 Hz, 1H), 3.80–3.77 (m, 1H), 3.71 (s, 2H), 3.62 (br s, 2H), 3.55 (m, 4H), 3.26–3.23 (m, 4H), 3.11 (br s, 2H), 3.05 (s, 6H), 2.43–2.38 (m, 2H), 1.96 (s, 3H); ^13^C NMR (125 MHz, DMSO-*d*_6_): *δ* 170.0, 167.8, 160.7, 154.6 (d, *J *= 246.2 Hz), 154.0, 152.8, 151.2, 144.6, 134.3 (d, *J *= 10.5 Hz), 133.7 (d, *J *= 9.0 Hz), 126.1, 123.1, 119.9 (d, *J *= 2.9 Hz), 114.1 (d, *J *= 2.4 Hz), 109.2, 109.0, 108.2, 106.6 (d, *J *= 26.0 Hz), 97.5, 71.6, 53.0, 51.2, 47.4, 47.3, 46.6, 41.4, 39.7, 38.5, 34.4, 24.3, 22.4; (+)-ESI-HRMS calc for C_35_H_43_FN_9_O_6_ [M+H]^+^: 704.3320, found 704.3309.

#### 5.1.10 (*S*)-*N*-((3-(3-Fluoro-4-(4-(3-(4-(((7-nitrobenzo[*c*][1,2,5]oxadiazol-4-yl)amino)methyl)-1*H*-1,2,3-triazol-1-yl)propyl)piperazin-1-yl)phenyl)-2-oxooxazolidin-5-yl)methyl)acetamide **30**

To a solution of azide **9** (50 mg, 0.12 mmol) and NBD fluorophore **26** (26 mg 0.12 mmol) in DMF (4 mL) was added a solution of CuSO_4_ (5 mol %, 1.5 mg, 0.006 mmol) and treated with aqueous sodium ascorbate (10 mol %, 2.4 mg, 0.012 mmol). The reaction was stirred vigorously at 50 °C for 16 h. Purification by column chromatography (Grace MPLC; C18 reverse phase with eluents 0.1% TFA in ACN/0.1% TFA in H_2_O) gave compound **54** (51.8 mg, 68%) as a orange solid. ^1^H NMR (600 MHz, DMSO-*d*_6_): *δ* 9.88 (t, *J *= 6.1 Hz, 1H), 8.53 (d, *J *= 8.9 Hz, 1H), 8.24 (t, *J *= 5.8 Hz, 1H), 8.16 (s, 1H), 7.51 (dd, *J *= 14.8, 2.3 Hz, 1H), 7.20 (dd, *J *= 8.8, 1.9 Hz, 1H), 7.12 (t, *J *= 8.9 Hz, 1H), 6.51 (d, *J *= 8.9 Hz, 1H), 4.78 (br s, 2H), 4.73–4.69 (m, 1H), 4.44 (t, *J *= 6.8 Hz, 2H), 4.08 (t, *J *= 9.0 Hz, 1H), 3.71–3.69 (m, 1H), 3.56 (br s, 2H), 3.40–3.35 (m, 4H), 3.19 (br s, 4H), 3.0 (br s, 2H), 2.24 (br s, 2H), 1.83 (s, 3H); ^13^C NMR (125 MHz, DMSO-*d*_6_): *δ* 170.0, 154.5 (d, *J *= 243.3 Hz), 154.0, 145.7, 144.5, 144.0, 142.7, 137.7, 134.2, 123.6, 119.9, 114.0 (d, *J *= 2.2 Hz), 106.5 (d, *J *= 26.0 Hz), 99.8, 71.6, 52.9, 52.0, 51.1, 47.2, 47.7, 41.3, 38.5, 24.2, 22.4; (+)-ESI-HRMS calc for C_28_H_33_FN_11_O_6_ [M+H]^+^: 638.2599, found 638.2602.

### 5.2 Biology

#### 5.2.1 Determination of MIC (minimum inhibitory concentration)

MICs were determined by a two-fold serial broth microdilution according to the recommendation of CLSI standards with an inoculum of 5 × 10^5^ cfu/mL. The compounds along with standard antibiotics were serially diluted twofold across the wells of 96-well non-binding surface plates (NBS, Corning). Standards ranged from 64–0.03 μg/mL, and the compounds from 128–0.06 μg/mL with final volumes of 50 μL per well. Gram-positive and Gram-negative bacteria were cultured in Mueller Hinton broth (MHB) (Bacto laboratories, Cat. No. 211443) at 37 °C overnight. A sample of each culture was then diluted 40-fold in fresh MHB broth and incubated at 37 °C for 2–3 h. The resultant mid-log phase cultures were diluted to the final concentration of 5 × 10^5^ cfu/mL, then 50 μL was added to each well of the compound containing 96-well plates. All the plates were covered and incubated at 37 °C for 24 h. MICs were the lowest concentration that showed no visible growth.

#### 5.2.2 Fluorescent microscopy of bacteria with fluorescent probes

An MRSA clinic isolate, Van A *E. faecium* ATCC 51559 and VRSA NARSA VRS3b were cultured using MHB broth at 37 °C. Fluorescent staining of bacteria was performed using a previously described method with slightly modifications.^62^ Mid-log phase cultures were incubated with 64 μg/mL of **29** and **30** at 37 °C for at least 1 h and 5 μL of each bacterial sample was used for preparing smears on glass slides. The smears were air-dried, fixed using 95% ethanol for 5 min and washed by PBS. Slides were then mounted using glycerol and covered with coverslips. A confocal microscope (Zeiss LSM 510) and software ZEN2009 was used for acquiring images, which were then processed using ImageJ software.

### 5.3 Modelling

The in silico docking was done using Schrödinger software package and its Induced Fit Docking module (Induced Fit Docking protocol 2013-2, Glide version 5.9, Prime version 3.2, Schrödinger, LLC, New York, NY, 2013). For the docking studies the crystal structure of ribosomal subunit 50S of *D. radiodurans* bound with linezolid (Pdb: 3DLL) was used.^55^ Default parameters for the induced fit docking workflow were used, using the bound linezolid molecule as ligand for the definition of the receptor site (box size of 40 Å around the ligand) and refinement of the receptor/pose complex including residues with 5 Å of the ligand. Ligands were prepared using the default Ligand Prep workflow, using the protonation state of the ligand at pH 7.0. The analysis of the different ligand orientations were limited to poses similar to the orientation of linezolid in the crystal structure.

## Supplementary data

Supplementary data 1 NMR spectra and HPLC chromatograms for **9**, **22**, **24**, and **27**–**30**.

### MOL files

The following ZIP file contains the MOL files of the most important compounds referred to in this article.

MOL files ZIP file containing the MOL files of the most important compounds in this article.
