# Supplementary material for: An azido-oxazolidinone antibiotic for live bacterial cell imaging and generation of antibiotic variants
Source: Bioorg Med Chem. 2014 Aug 15;22(16):4490–8. doi: 10.1016/j.bmc.2014.05.054 (PMC4141890; doi:10.1016/j.bmc.2014.05.054)
Supplement: Supplementary data 1 — NMR spectra and HPLC chromatograms for 9, 22, 24, and 27–30. [file mmc1.doc]

Supplementary Material:

azidopropyl 4-methylbenzenesulfonate 22

^1^H NMR (600 MHz, CDCl_3_)

azidopropyl 4-methylbenzenesulfonate 22

^13^C JMOD NMR (125 MHz, CDCl_3_)

1. (*S*)-*N*-((3-(4-(4-(3-azidopropyl)piperazin-1-yl)-3-fluorophenyl)-2-oxooxazolidin-5-yl)methyl)-acetamide 9

LCMS

(*S*)-N-((3-(4-(4-(3-azidopropyl)piperazin-1-yl)-3-fluorophenyl)-2-oxooxazolidin-5-yl)methyl)-acetamide 9

^1^H NMR (600 MHz, CD_3_OD)

(*S*)-N-((3-(4-(4-(3-azidopropyl)piperazin-1-yl)-3-fluorophenyl)-2-oxooxazolidin-5-yl)methyl)-acetamide 9

^13^C JMOD NMR (125 MHz, CD_3_OD)

1. 2-(7-(dimethylamino)-2-oxo-2H-chromen-4-yl)-*N*-(prop-2-yn-1-yl)acetamide 24

LCMS

2-(7-(dimethylamino)-2-oxo-2H-chromen-4-yl)-*N*-(prop-2-yn-1-yl)acetamide 24

^1^H NMR (600 MHz, DMSO-*d*_6_)

2-(7-(dimethylamino)-2-oxo-2H-chromen-4-yl)-*N*-(prop-2-yn-1-yl)acetamide 24

^13^C JMOD NMR (125 MHz, DMSO-*d*_6_)

1. (*S*)-*N*-((3-(3-fluoro-4-(4-(3-(4-phenyl-1H-1,2,3-triazol-1-yl)propyl)piperazin-1-yl) phenyl)-2-oxooxazolidin-5-yl)methyl)acetamide 27

LCMS

(*S*)-N-((3-(3-fluoro-4-(4-(3-(4-phenyl-1H-1,2,3-triazol-1-yl)propyl)piperazin-1-yl) phenyl)-2-oxooxazolidin-5-yl)methyl)acetamide 27

^1^H NMR (600 MHz, DMSO-*d*_6_)

(*S*)-*N*-((3-(3-fluoro-4-(4-(3-(4-phenyl-1H-1,2,3-triazol-1-yl)propyl)piperazin-1-yl) phenyl)-2-oxooxazolidin-5-yl)methyl)acetamide 27

^13^C JMOD NMR (125 MHz, DMSO-*d*_6_)

1. (*S*)-*N*-((3-(3-fluoro-4-(4-(3-(4-(hydroxymethyl)-1H-1,2,3-triazol-1-yl)propyl)piper -azin-1-yl)phenyl)-2-oxooxazolidin-5-yl)methyl) acetamide 28

LCMS

(*S*)-N-((3-(3-fluoro-4-(4-(3-(4-(hydroxymethyl)-1H-1,2,3-triazol-1-yl)propyl)piper -azin-1-yl)phenyl)-2-oxooxazolidin-5-yl)methyl) acetamide 28

^1^H NMR (600 MHz, DMSO-*d*_6_)

(*S*)-*N*-((3-(3-fluoro-4-(4-(3-(4-(hydroxymethyl)-1H-1,2,3-triazol-1-yl)propyl)piper -azin-1-yl)phenyl)-2-oxooxazolidin-5-yl)methyl) acetamide 28

^13^C JMOD NMR (125 MHz, DMSO-*d*_6_)

1. (*S*)-*N*-((1-(3-(4-(4-(5-(acetamidomethyl)-2-oxooxazolidin-3-yl)-2-fluorophenyl) piperazin-1-yl)propyl)-1H-1,2,3-triazol-4-yl)methyl)-2-(7-(dimethylamino)-2-oxo-2H-chromen-4-yl)acetamide 29

LCMS

(*S*)-*N*-((1-(3-(4-(4-(5-(acetamidomethyl)-2-oxooxazolidin-3-yl)-2-fluorophenyl) piperazin-1-yl)propyl)-1H-1,2,3-triazol-4-yl)methyl)-2-(7-(dimethylamino)-2-oxo-2H-chromen-4-yl)acetamide 29

^1^H NMR (600 MHz, DMSO-*d*_6_):

(*S*)-*N*-((1-(3-(4-(4-(5-(acetamidomethyl)-2-oxooxazolidin-3-yl)-2-fluorophenyl) piperazin-1-yl)propyl)-1H-1,2,3-triazol-4-yl)methyl)-2-(7-(dimethylamino)-2-oxo-2H-chromen-4-yl)acetamide 29

^13^C JMOD NMR (125 MHz, DMSO-*d*_6_)

1. (*S*)-*N*-((3-(3-fluoro-4-(4-(3-(4-(((7-nitrobenzo[c][1,2,5]oxadiazol-4-yl)amino) methyl)-1H-1,2,3-triazol-1-yl)propyl)piperazin-1-yl)phenyl)-2-oxooxazolidin-5-yl)methyl)acetamide 30

LCMS

(*S*)-*N*-((3-(3-fluoro-4-(4-(3-(4-(((7-nitrobenzo[c][1,2,5]oxadiazol-4-yl)amino) methyl)-1H-1,2,3-triazol-1-yl)propyl)piperazin-1-yl)phenyl)-2-oxooxazolidin-5-yl)methyl)acetamide 30

^1^H NMR (600 MHz, DMSO-*d*_6_):

(*S*)-*N*-((3-(3-fluoro-4-(4-(3-(4-(((7-nitrobenzo[c][1,2,5]oxadiazol-4-yl)amino) methyl)-1H-1,2,3-triazol-1-yl)propyl)piperazin-1-yl)phenyl)-2-oxooxazolidin-5-yl)methyl)acetamide 30

^13^C JMOD NMR (125 MHz, DMSO-*d*_6_)
